# Inputs for universal health coverage: a methodological contribution to finding proxy indicators for financial hardship due to health expenditure

**DOI:** 10.1186/s12913-014-0577-2

**Published:** 2014-11-25

**Authors:** Priyanka Saksena, Thomas Smith, Fabrizio Tediosi

**Affiliations:** Swiss Tropical and Public Health Institute, University of Basel, Socinstrasse 57, 4051 Basel, Switzerland; Centre for Research on Health and Social Care Management (CERGAS), Università Bocconi, Milan, Italy

**Keywords:** Universal health coverage, Financial hardship, Financial burden, Financial risk protection, Catastrophic health expenditure, Impoverishment, Out-of-pocket payments, Health payments, Health expenditure

## Abstract

**Background:**

Universal health coverage is high on national health agendas of many countries at the moment. Absence of financial hardship is a key component of universal health coverage and should be monitored regularly. However, relevant household survey data, which is traditionally needed for this analysis is not frequently collected in most countries and in some countries, has not been collected at all. As such, proxy indicators for financial hardship would be very useful.

**Methods:**

We use data from the World Health Survey and use multi-level modeling with national and household level characteristics to see which indicators have a consistent and robust relationship with financial hardship. To strengthen the validity of our findings, we also use different measures of financial hardship.

**Results:**

There are several household level characteristics that seem to have a consistent relationship with financial hardship. However there is only one strong candidate for a proxy indicator at the national level– the share of out-of-pocket payments in total health expenditure. Additionally, the Gini coefficient of total household expenditure was also correlated to financial hardship in most of our models.

**Conclusion:**

The national level indicators related only weakly to the risk of financial hardship. Hence, there should not be an over-reliance on them and collecting good quality household survey data is still a superior option for monitoring financial hardship.

**Electronic supplementary material:**

The online version of this article (doi:10.1186/s12913-014-0577-2) contains supplementary material, which is available to authorized users.

## Background

Universal health coverage is currently high on the national health agendas of many low and middle income countries, as well as at the global level. The objectives of universal health coverage are to ensure access to quality health services whenever these are needed, and to avoid financial hardship due to the use of health services [1,2]. Health services contribute to good health, but the payments required to obtain these services force some households to face severe financial difficulties. Thus, measuring to what extent a health system protects households from financial loss due to paying for health services is critical for assessing the overall system.

The incidence of catastrophic health expenditure is one of the measures used to assess financial burden from health payments [3]. The indicator was developed with the thinking that people’s out-of-pocket payments (OOP) should be related to ability or capacity to pay. The basic idea of catastrophic health expenditures is that households that have an excessive burden due to health payments relative to their capacity to pay are thought to have reduced other basic necessary spending in order to pay for health. Although there are different ways of constructing the indicator, the guiding principle behind it remains the same. In the past decade, percentage of households facing catastrophic health expenditure has been commonly used to monitor and evaluate financial protection in a health system.

Another measure to assess the financial burden is impoverishment due to health payments [4]. The impoverishment indicator is cemented in the economics literature on the role of poverty [5-8]. Similarly to catastrophic health expenditure, whereas there are various ways of constructing the poverty line in relation to defining impoverishment, the guiding principles of the indicator transcend these differences [6,9]. Additionally, like catastrophic health expenditure, impoverishment has been widely used to document and draw attention to the financial protection situation in countries.

The estimation of these indicators requires household survey data which represent the whole country and contain information on total household and health expenditure. This type of survey exists in many countries. A widely cited study managed to report the indicator in nearly 90 countries [10]. However, for some countries the data were based on surveys from the early 1990s. Indeed, very few countries conduct household surveys annually [11]. A three to five years interval for conducting surveys is common in some countries, while surveys are even less frequent in other countries.

In the meantime, many countries have already instituted reforms to move towards universal health coverage, while others are in the process of determining what path may be best suited for them [12-16]. As countries attempt to move towards universal coverage, there is a need for monitoring progress. Monitoring the financial burden from health payments has been proposed as a key target for universal health coverage achievements by the World Bank and WHO [17]. This essential task is challenging both at the national level as well as the international level as new data are needed, while existing data need to be used more effectively. While strengthening the information system, proxy indicators which allow rapid evaluation of financial protection are needed in order to assess and if necessary, modify policies to move towards universal coverage.

In this paper, we attempt to examine which proxy indicators may be best suited to gauge the level of financial protection in a country as measured by incidence of catastrophic health expenditure and impoverishment, which have been used extensively in previous literature. The choice of potential proxies is limited in itself and the most reliable candidates are data on health systems financing, which are available and updated annually in National Health Accounts exercise [18]. Generally, there has been a lot of interest in these indicators because they provide a standard interpretation of complex aspects of health systems and allow for cross-country as well as time-series comparisons. Additionally, previous studies have documented the relationship between health systems financing indicators and financial risk protection [10,19].

In this study, we add to this literature by employing a methodological framework to test which indicators are best suited to measure financial protection in the absence of household survey data. Using data from the World Health Survey, we model the incidence of catastrophic health expenditure and impoverishment against household level characteristics as well as national-level health systems indicators through a cross-country multi-level approach. This research can guide policymakers on how to increase financial protection, as well as what types of intermediate indicators are pertinent to monitoring progress towards universal health coverage.

## Methods

### Data source

The household level data used in this study were from the World Health Surveys (WHS) from 51 countries [20]. The data was collected in the 2002–2003 period. All WHS data are nationally representative, with sample size from 659 to 38424 households. The sample size exceeded 1000 households in 90% of the national surveys used. The sample size in each country is presented in Additional file 1: Table S1. The standardized questionnaires provide in-depth information on household characteristics and health expenditure. All the expenditure variables including health and non-health spending were reported based on a 4-week recall period in the survey. We combined the household information with country-level indicators of health systems financing from the National Health Accounts database [21]. In terms of household level data, it should be noted that as this study relied solely on secondary anonymized survey data that is available in the public domain, approval from an ethics committee was not sought. No primary data was collected for this study.

### Outcome variables

The first measure of financial burden, catastrophic health expenditure from OOP has been used in many previous studies [22-25]. It aims to capture how when households have to spend high proportions of their available resources on OOP. In this study, we defined catastrophic health expenditure using the two most commonly used methods, both of which are based on food expenditure. In the first method (1A), which is used by the WHO, all household expenditure exceeding a particular food expenditure threshold is considered to be non-subsistence expenditure. Under this method, a household is defined as facing catastrophic health expenditure if its health spending exceeds 40% of its non-subsistence expenditure. The definition of subsistence expenditure, the converse of non-subsistence expenditure, is related to basic food expenditure. The commonly used methodology is described in detail in other papers [26]. In the second method (1B), we define catastrophic health expenditure as occurring when a household spends more than 40% of its non-food expenditure on health, which has also be commonly employed [27,28]. For these two methods, we also considered lower thresholds of 20%. Additionally, sensitivity analysis was performed with additional thresholds of 10% and 30%.

The second measure of financial burden, impoverishment due to OOP, is also a well-defined concept. The idea behind it is to measure how many households are pushed below the poverty line due to OOP. Similarly to the incidence of catastrophic health expenditure, impoverishment due to OOP is also a binary variable. However, there are different ways of measuring at what constitutes as the poverty line. In this paper, we use 3 different poverty lines. The first two lines are the World Bank defined international poverty lines of Int $1.25 and Int $2 per person per day [29]. These are respectively called methods 2A and 2B. In addition to these absolute poverty lines, we also present a relative poverty line based on food expenditure, which has been promoted by the WHO [9,30] – this method is 2C. The poverty line used in this method is the same as the food expenditure threshold that was identified for calculating catastrophic health expenditure in method 1A.

We also explored one other way of defining what constitutes financial burden from health payments. Generally, the financial burden is calculated for all households. However, there is some logic is limiting this to just households with health expenditures, which may be considered as a proxy for health services use, since financial hardship is an outcome restricted to households that are able to access care in the first place [31,32]. As such in this analysis, we also separately measured the financial burden among both all households and among just households with health expenditures.

The different outcome variables are summarized in Table 1.

**Table 1:** **Different outcome variables modelled**

**Models**	**Definition of a household with financial hardship**
	Catastrophic health expenditure
**1A - 20%**	OOP ≥20% of non-subsistence expenditure
**1A - 40%**	OOP ≥40% of non-subsistence expenditure
**1B - 20%**	OOP ≥20% of non-food expenditure
**1B - 40%**	OOP ≥40% of non-food expenditure
	Impoverishment
**2A**	Total expenditure ≥ Int$ 1.25 * Household size & Total expenditure – OOP < Int$ 1.25 * Household size
**2B**	Total expenditure ≥ Int$ 2.0 * Household size & Total expenditure – OOP < Int$ 2.0 * Household size
**2C**	Total expenditure ≥ Relative food-based poverty line * Adjusted household size & Total expenditure – OOP < Relative food-based poverty line * Adjusted household size

### Co-variates

The household level co-variates were: whether the household had members under 5 years of age; whether the household had members over 60 years of age; whether the household had any disabled members; the education level of the household head for completion of primary school and for completion of secondary school or higher; the sex of the household head; whether the household lives in an urban area; whether the household had health insurance as reported in the WHS survey; and quintiles based on total household expenditure. These household expenditure quintiles were derived separately for each country. The choice of these covariates was due to the availability in the WHS survey as well as previous evidence of their linkage with financial burden [26,33-35].

The country level variables were: total health expenditure per capita in international dollars (adjusted for Purchasing Parity Power, PPP); out-of-pocket payments as a share of total health expenditure; government expenditure on health as a share of total government expenditure; and binary variables for whether a country’s prepayment was channelled primarily through a tax-based or social health insurance-based system, or through a combination of the two. A country’s prepayment was defined as being channelled primarily through a tax-based system if 60% or more of its prepayment was tax-based. A similar approach was taken for social health insurance-based systems, while a mixed system was defined when neither tax-based or social health insurance-based prepayment exceeded 60% of total prepayment in a country. We also included the Gini coefficient of expenditure as a country level variable, which was derived from total expenditure in the WHS surveys for each country. The Gini coefficient will be higher for countries that are considered to have a more equal distribution of expenditure (i.e. less inequality). The choice of these covariates was driven by their availability for the countries in the WHS as well as previous evidence of their linkage with financial burden [10,19,36]. Other national level covariates were also initially included, but they were excluded from the final models presented here because of a lack of correlation with the outcomes. These included the density of different types of health personnel, distribution of health personnel within a country and hospital beds in the country.

### Models

In order to understand the levels and variation of financial burden within as well as across countries, and across a range of covariates, multilevel regression techniques were used [37]. These techniques allow for a much more rigorous examination of national level factors and their influence on the financial burden while simultaneously examining the effects of household level factors. As such, this technique is particularly well suited to determining what national level indicators may be closely linked to financial burden, which can in turn be used to monitor progress towards universal coverage in the absence of household level data.

To explore the factors that may be associated with financial burden, generalized linear mixed-effects model was used. Mixed-effects models allow for nesting of households within countries, thus providing more robust evidence on the effect of country-level variables as discussed earlier. This is done partitioning the variance of observed variables into individual and country levels. We used a random-coefficients model, which allows for variation in the estimates of coefficients of the household level effects. The linear component of the model takes on the form:

$$ {\mathrm{y}}_{\mathrm{ij}} = \mathrm{k} + \beta {0}_{\mathrm{j}} + \beta {1}_{\mathrm{j}}{\mathrm{x}}_{\mathrm{ij}} + \beta 2{\mathrm{z}}_{\mathrm{j}} + {\mathrm{e}}_{\mathrm{ij}} $$

Where *i* are households within countries, *j* are countries. Y_ij_ is the outcome variable for a household *i* in a country *j*. x_ij_ are household level variables. However, unlike conventional models, the coefficients for these household specific variables, β1 are allowed to vary by country (i.e. each country *j* will have its own set of parameter estimates of β1). Further, z_i_ are country-level variables, whose effects on y_ij_ are estimated through coefficients β2, which do not vary. Additionally, k is the overall intercept in the model, while β0_j_ are country-specific intercepts. Finally, e_ij_ is the error in the estimating equation.

However, as our outcome variables for financial burden are binary, we cannot use a linear regression model. Instead, we use a generalized linear mixed-model, which allows for probability distribution other than a normal distribution using a link function to transform the outcome variable. Assuming a binomial distribution, we transform the outcome variables using a logit link function. The parameters for this are estimated by the following equations:

$$ \begin{array}{l}\mathrm{g}\left(\mathrm{E}\left({\mathrm{y}}_{\mathrm{ij}}\right)={\upmu}_{\mathrm{ij}}\right) = \upbeta {0}_{\mathrm{j}} + \upbeta {1}_{\mathrm{j}}{\mathrm{x}}_{\mathrm{ij}} + \upbeta 2{\mathrm{z}}_{\mathrm{j}}\kern1.75em \mathrm{where}\kern0.24em g\kern0.24em \mathrm{is}\ \mathrm{the}\ \mathrm{logit}\ \mathrm{link}\ \mathrm{function}\ \mathrm{specified}\ \mathrm{b}\mathrm{y}:\\ {} \ln \left({\upmu}_{\mathrm{ij}}/\left(1 - {\upmu}_{\mathrm{ij}}\right)\right)\end{array} $$

It should be noted that the coefficients from this equation can be interpreted in same way as coefficients from a standard logistic regression. The analysis for this paper was carried out using the R statistical software. The multi-level models were run using the lme4 package.

## Results

### Descriptive statistics

Table 2 presents the descriptive statistics for the national level variables in the model. As would be expected, there is a high degree of variance for many of the variables, particularly per capita total health expenditure.

**Table 2:** **Descriptive statistics for national level variables**

**National level variables**	**Mean**	**Standard deviation**
**Per capita total health expenditure (PPP $)**	368.0	435.5
**Government health expenditure as % of total government expenditure (%)**	9.7	3.6
**Out-of-pocket payments as a % of total health expenditure (%)**	41.5	19.2
**Gini coefficient of total household expenditure**	0.443	0.104
**Percentage of countries with predominately tax-funded prepayment (%)**	76.5	42.8
**Percentage of countries with predominately social health insurance-funded prepayment (%)**	15.7	36.7
**Percentage of countries with predominately mixed financing of prepayment (%)**	7.8	27.2

Table 3 contains the descriptive statistics for the household level variables in the model, which are all binary variables. The variables with the most variation across countries is households with heads who have completed secondary or higher education and reports of insurance coverage. Other notable characteristics are that only around 1 in 5 households are headed by women and only around 1 in 9 households had members who with disabilities. Around half the households in the sample lived in urban areas and only around 1 in 4 households reported insurance coverage.

**Table 3:** **Descriptive statistics for household level variables**

**Variable**	**Overall proportion of households**	**95 % Confidence Interval**		**Overall standard deviation**	**Standard deviation between countries**	**Standard deviation within countries**
**Household member under 5**	0.338	0.336	0.340	0.473	0.151	0.456
**Household member over 60**	0.308	0.306	0.310	0.462	0.113	0.450
**Household head with primary education**	0.420	0.418	0.422	0.494	0.184	0.455
**Household head with secondary or higher education**	0.219	0.218	0.221	0.414	0.247	0.366
**Male household head**	0.800	0.799	0.802	0.400	0.110	0.390
**Urban residence**	0.495	0.494	0.497	0.500	0.221	0.449
**Insurance coverage reported**	0.253	0.250	0.250	0.435	0.381	0.308
**Disabled household member**	0.112	0.110	0.113	0.315	0.036	0.313

Table 4 shows the mean incidence of catastrophic health expenditure and impoverishment across different countries using the different methods described in the methodology section. Overall, the incidence of catastrophic health expenditure using the non-food methodology is higher than when non-subsistence expenditure is used as would be expected. Similarly, the incidence is considerably higher when only households with positive OOP are considered, once again as would be expected. The incidence of impoverishment is a fraction of the incidence of catastrophic health expenditure irrespective of the measure of impoverishment. But there is also a lot of variation in the financial burden measured through these indicators across different countries. As would be expected due to the smaller sample size, the 95% confidence interval for these values is wider when only households who had any OOP were considered.

**Table 4:** **Descriptive statistics for outcome variables**

	**All households (N = 241,706)**				**Households with OOP > 0 (N = 138,167)**			
	**Mean**	**Standard deviation**	**95 % Confidence interval**		**Mean**	**Standard deviation**	**95 % Confidence interval**	
**Catastrophic health expenditure**								
**1A – 20%**	0.300	0.458	0.299	0.302	0.525	0.499	0.523	0.528
**1A – 40%**	0.171	0.376	0.169	0.172	0.299	0.458	0.296	0.301
**1B – 20%**	0.372	0.483	0.370	0.374	0.591	0.492	0.588	0.593
**1B – 40%**	0.236	0.425	0.235	0.238	0.353	0.478	0.351	0.356
**Impoverishment**								
**2A**	0.035	0.183	0.034	0.036	0.061	0.239	0.060	0.062
**2B**	0.045	0.208	0.044	0.046	0.079	0.270	0.078	0.081
**2C**	0.053	0.224	0.052	0.054	0.092	0.290	0.091	0.094

We also examined the correlation between the outcome variables and the co-variates. The correlations of the household level variables were tested using a Spearman test. The results are presented in the Additional file 1: Table S2. Overall, these results show a large consistency in the direction and significance of the relationship between many of the household variables and the outcome variables. It is also interesting to note that sign of the male household head variable changes between the models with all households and the models including just households that have made OOP. This is likely to be due to a higher utilization rate for households with male heads.

We also plotted the mean incidence of catastrophic health expenditure at various thresholds against national level variables that are continuous. The results are presented in the Additional file 1: Figure S1. There are no clear patterns that emerge in these plots.

### Main results

The results from the different multi-level random-coefficients regressions models are shown in Table 5. The table shows the regression coefficients, alongside the significance of the variable in the model. Additional regressions with 10% and 30% thresholds for catastrophic health expenditure were also performed and the results of them are presented in Additional file 1: Table S3.

**Table 5:** **Regression models’ results**

	**All households**							**Households with OOP >0**						
	**1A - 20%**	**1A - 40%**	**1B - 20%**	**1B - 40%**	**2A**	**2B**	**2C**	**1A - 20%**	**1A - 40%**	**1B - 20%**	**1B - 40%**	**2A**	**2B**	**2C**
**National level variables**														
**Per capita total health expenditure (PPP $)**	−0.025	0.022	0.041	−0.007	−0.544*****	−0.497*****	0.024	0.057	0.014	0.01	−0.104	−0.476****	−0.425****	0.032
**Government health expenditure over total government expenditure (%)**	0.310	−1.648	0.036	0.05	10.289*****	8.311**	1.921	1.780	1.308	2.356	3.54	10.837**	6.88	8.689**
**Gini coefficient**	0.747	1.446**	1.704****	2.242*****	3.355*****	0.227	2.458***	1.360**	1.686**	1.288*	2.118****	4.379****	0.767	2.933**
**Predominately tax-funded financing (0,1)**	0.176	−0.046	0.069	−0.285	−0.296	0.251	0.178	−0.052	−0.191	−0.062	−0.11	−0.212	0.849	0.61
**Predominately mixed financing (0,1)**	0.103	0.179	0.019	−0.211	−0.642	−0.011	−0.717*	0.031	0.048	−0.05	−0.071	−0.628	0.757	−0.822
**Out-of-pocket payments as a share of total health expenditure (%)**	1.908*****	2.504*****	2.155*****	2.823*****	2.423*****	2.457***	3.638*****	2.000*****	2.261*****	2.671*****	2.598*****	1.98**	1.798*	3.44*****
**Household level variables**														
**Household member under 5 (0,1)**	0.208*****	0.228*****	0.170*****	0.146*****	0.408*****	0.310*****	0.216*****	−0.044	0.025	−0.037	0.008	0.239*****	0.205*****	0.047
**Household member over 60 (0,1)**	0.355*****	0.364*****	0.376*****	0.378*****	0.202*****	0.276*****	0.325*****	0.329*****	0.323*****	0.356*****	0.345*****	0.162*****	0.186*****	0.252*****
**Household head with primary education (0,1)**	−0.007	−0.135*****	−0.070****	−0.193*****	−0.055	−0.063	−0.103***	−0.137*****	−0.251*****	−0.151*****	−0.251*****	−0.174*****	−0.142*****	−0.188*****
**Household head with secondary or higher education (0,1)**	−0.174*****	−0.381*****	−0.217*****	−0.397*****	−0.227**	−0.233****	−0.298*****	−0.305*****	−0.493*****	−0.338*****	−0.491*****	−0.325*****	−0.309*****	−0.376*****
**Male household head (0,1)**	−0.064****	−0.060**	−0.064****	−0.058**	0.068	0.090***	−0.081*	−0.133*****	−0.095*****	−0.12*****	−0.085****	0.044	0.059*	−0.134****
**Urban residence (0,1)**	−0.167*****	−0.316*****	−0.231*****	−0.348*****	−0.361*****	−0.299*****	−0.304*****	−0.186*****	−0.333*****	−0.2*****	−0.33*****	−0.394*****	−0.31*****	−0.289*****
**Insurance coverage reported (0,1)**	−0.105***	−0.197*****	−0.134****	−0.232*****	−0.642*****	−0.319*****	−0.236***	−0.130*****	−0.198*****	−0.141*****	−0.206*****	−0.514*****	−0.3*****	−0.22**
**Disabled household member (0,1)**	0.554*****	0.549*****	0.496*****	0.437*****	0.537*****	0.590*****	0.504*****	0.311*****	0.322*****	0.298*****	0.283*****	0.318*****	0.397*****	0.28*****
**Quintile 2 (0,1)**	0.189*****	0.099***	−0.015	−0.147****	0.061	0.473	3.773*****	−0.335*****	−0.301*****	−0.289*****	−0.259*****	−0.417	0.285	3.21*****
**Quintile 3 (0,1)**	0.146**	0.011	−0.003	−0.153**	−0.089	0.707	4.683*****	−0.660*****	−0.579*****	−0.437*****	−0.355*****	−0.581	0.448	4.087*****
**Quintile 4 (0,1)**	0.055	−0.117**	0.046	−0.106	−0.218	1.181*	3.667*****	−1.030*****	−0.883*****	−0.572*****	−0.401*****	−0.763	0.773	2.921*****
**Quintile 5 (0,1)**	0.060	0.088	0.097	0.063	−0.401	1.207*	3.064*****	−1.210*****	−0.794*****	−0.697*****	−0.318*****	−1.113**	0.618	2.121***

*p-value <0.1.

**p-value <0.05.

***p-value <0.01.

****p-value <0.005.

*****p-value <0.001.

Several household level characteristics have a consistent association with the outcome indicators of financial hardship. Households with members over 60 years of age or disabled members had a higher incidence of financial hardship in all the models. On the other hand, there was no significant association between having children under 5 years of age and the incidence of catastrophic expenditure when only households with OOP are considered, while there is a strong and consistent association when all households are considered. This is likely to be related differences in utilization of health services for households with children under 5 years of age and possibly with free care policies for children.

In terms of other socio-economic characteristics such as households living in urban areas, households with heads who have completed secondary or higher education and households who reported health insurance coverage have a lower incidence of financial hardship. Additionally, as would be expected, households in higher quintiles had a lower incidence when only households with OOP spending were considered in most of the models. The only exceptions were the models with impoverishment.

We found that most of the indicators of health systems financing had no consistent relationship with the incidence of financial hardship. The share of government health expenditure to total government expenditure, whether a country’s prepayment for health relied primarily on a tax-based or social health insurance-based system, or through a combination of the two had no consistent relationship with our indicators of financial hardship. Total health expenditure per capita in PPP dollars only had a negative significant relationship in models 2A and 2B.

In fact, only the share of out-of-pocket payments in total health expenditure had a consistent positive and significant association with financial hardship. The overall level of inequality in a country, represented here by the Gini coefficient of total household expenditure, seems to have a positive association with financial hardship. However, interestingly, this relationship is not significant in models with the lower thresholds of catastrophic health expenditure and model 2B when all households are considered.

### Model diagnostics

For each of the models and thresholds presented in this section, simpler specifications were attempted before selecting the random coefficients model specified in the methodology section. Firstly, a random intercept model for country effects with just national level variables was tried, followed by a random intercept model for country effects with just household level variables. The third model was a random intercept model for country effects with both national and household variables. Finally, the random coefficients model discussed in the methodology was tried, with varying coefficients for household variables at the country level alongside national level variables and random country intercepts as well as an overall global intercept. The latter model was superior in terms of fit as compared to the 3 other models described for all the specifications using the criteria of AIC, BIC and log likelihood. Overall, the models considering just households with out-of-pocket expenditures had better fits than the models with all households on the basis of the AIC, BIC and log likelihood. The correlation coefficients for the national level variables were also checked and not found to be problematic. This information is presented in Additional file 1: Tables S4 and S5.

## Discussion

The results from this study reinforce previous findings that both household characteristics and health system indicators are associated with financial hardship [34,38-42]. At the household level we found that households that live in rural areas, that are poorer, that have less education and lack health insurance face more financial hardship due to OOP. Additionally, demographic and health characteristics such as the presence of elderly and disabled members also renders households more susceptible to financial hardship from OOP. Policymakers in all countries still need to improve financial risk protection for these at-risk households. Many of these types of households are also likely to have lower health status and be more vulnerable in other domains as well [43]. As such, the health system can play an important role in decreasing overall inequality by ensuring that these types of households benefit fully from initiatives to move towards universal health coverage.

At the national level, some health systems characteristics influence the incidence of financial hardship. We found that the degree of reliance on out-of-pocket payments to finance health is associated with higher levels of catastrophic health expenditure across all models, which is consistent with previous studies [10]. We also found a significant relationship between financial hardship and income inequality in most models, which may be related to the effect of inequality on price levels.

However, we found no consistent relationships between other national variables and financial hardship. There was no direct relationship between whether a system is tax-based or social health insurance-based and financial hardship and strikingly, higher priority for health in government budgets was also not associated with lower incidence of financial hardship. The level of total health expenditure per capita was also only significant in 2 of the impoverishment models. The lack of significant relationship between these aspects and financial hardship is quite pertinent. But it is worth highlighting that dependent variables in this paper are binary indicators of financial hardship. Ability to access or use services was outside the scope of this particular analysis. It is very likely that these important national characteristics may influence the ability to access services in the first place. Indeed, the availability of services is likely to be governed by aspects such as the level of funding in the system and priority placed on health by governments. Nonetheless, it is notable the degree of financial hardship suffered by households does not seem to be directly affected by these factors.

The strongest candidate for national level proxy indicator of financial hardship is out-of-pocket payments as a share of total health expenditure, followed by the level of inequality of overall expenditure. Both these indicators are continuous and have ordinal properties. As such, they seem to well suited to be proxy indicators. At the household level, some of the characteristics we modelled are not particularly well suited to act as proxy indicators of financial hardship, such as household expenditure quintiles. Other characteristics such the percentage of households with elderly members may be difficult to influence in any ethical or logical way. The household level characteristics that could have some merit as proxy indicators are household heads with secondary or higher education and the percentage of households living in urban areas. However, exactly how these characteristics influence financial hardship is unclear and they may just be linked to the influence of overall socio-economic status on financial hardship. If they are linked to overall socio-economic status, this would be problematic since overall socio-economic status is likely to be distributional variable within each country, much like household expenditure quintiles.

Households who have reported insurance coverage could also seem interesting to consider at first as a potential proxy indicator for financial hardship. However, this is a tricky indicator to interpret. In countries like the United Kingdom, what we typically consider insurance does not play a large role in the health system. On the other hand, in Germany for example, the mechanism of coverage is one that is typically called insurance. As such, a household in the United Kingdom may report that they do not have health insurance, whereas a household in Germany would report that they do. But entitlements to services and financial risk protection is not fundamentally different in the United Kingdom or Germany. As such, an indicator of households with insurance is difficult to interpret at the international level and hence should not be used as proxy indicator for financial hardship.

There are also some interesting findings of this study with regards to the choice of outcome indicators for financial hardship. For example, it is interesting to note at the 10% threshold for catastrophic health expenditure, richer households have a higher risk of facing catastrophic health expenditure when all households are considered. This is a relevant commentary on the construction of catastrophic health expenditure which is a measure of spending on OOP relative to other expenditures and on the income elasticity of health expenditure. Richer households are likely to spend a greater proportion of their disposable income on health. This is likely to be reflected when lower thresholds are used for constructing the measure of catastrophic health expenditure. On the other hand, higher thresholds in the construction of catastrophic health may be more reflective of real financial burden, rather than just discretionary spending.

Similarly, outcome indicators of financial hardship based on impoverishment are also sensitive to the choice of poverty line. In this paper, we try to overcome this issue by presenting multiple poverty lines. But some of the results are still indicative of the potential bias in impoverishment headcount indicators. For example, richer households are more likely to be impoverished under model 2C, while household quintiles are particularly significant in models 2A or 2B. This misleading result is due to the fact that almost all Quintile 1 households are classified as being under the poverty line irrespective of health payments with the relative poverty line used in method 2C. But far fewer households in Quintile 2 and above are under the poverty line irrespective of health payments in this model. On the other hand with the absolute poverty lines of models 2A and 2B, in high-income countries, almost no households are under the poverty line irrespective of health payments, while in many low-income countries, almost all households are under the poverty line irrespective of health payments. Naturally, this considerably limits the potential variance of the outcome variable, which only looks at households that are not under the poverty line before health payments. This limited variance of the outcome variable will also influence the significance of the national level variables in our models. Overall, relative poverty may perform better when there is a wide range of countries in the sample, like in our case. However, it may not be sufficient to monitor just the impoverishment headcount to understand the degree of financial hardship – a measure of the difference in depth of poverty due to health payments may also be needed.

Although interesting findings emerge from this study, we need to be aware of the limitations. Health expenditure from WHS may be high compared to non-health household surveys. The high levels of health expenditure found in the WHS survey have been commented on by various authors [11,44,45]. However, there is no evidence that there were any systematic differences in the reporting of health expenditure in different countries as well as across different groups of people. In terms of frameworks, in this study we only considered health systems financing parameters and their relationship with the common indicators of financial hardship. We are, of course, conscious that other aspects of health systems also play an important role in governing the financial burden from health payments in a country, for example the density of hospitals versus health centres and medicines’ prices. Additionally, non-health systems characteristics such as access to social networks in times of financial need may also influence financial hardship. However, due to the lack of consistent data for this type of information across the countries in the dataset, we were not able to directly control for these factors in our analysis. Finally, the WHS dataset is a cross-sectional dataset and as such, it has some inherent limitations with regards to its ability to inform analysis concerned with time-series implications. However, we think that its use for this study is appropriate because we try to model the generic characteristics that are linked to financial hardship. By virtue of being generic, we think a snapshot in time will in fact also reflect the path countries take on the road to improved financial risk protection. Our choice to use the WHS for this purpose is also driven by the fact that unfortunately there are no multi-country panel surveys that are consistent enough to be used for in a multilevel model looking at financial hardship.

## Conclusion

In this paper we use a robust methodology to examine which indicators may be better suited as proxies for the incidence of financial hardship. We found that certain indicators are better suited for this purpose than others.

The key candidate for a proxy indicator of financial hardship is the share of out-of-pocket payments in total health expenditure. Alongside out-of-pocket payments in total health expenditure, it may also be useful to monitor the evolution of the overall level of inequality in a country using the Gini coefficient of household expenditure. Decreased reliance on out-of-pocket payments to finance health and decreased inequality in expenditures in general seem to indicate better levels of financial risk protection. This is an important finding for policymakers in countries that are interested in monitoring their progress towards universal health coverage.

However, that is not to say that these indicators are perfect representations of complex realities. For example, the share of out-of-pocket payments in total health expenditure is limited in many ways. This national level indicator cannot tell us anything about the distribution of out-of-pocket payments in a country. The distribution of out-of-pocket payments is essential to answering important policy questions that cannot be adequately addressed by one or two national level indicators. Therefore, whereas these proxy indicators may serve an important role in monitoring progress towards universal health coverage, their inherent limitations should not be overlooked.

Similarly, of course, our study also does not imply that the other indicators that were not found to be associated with financial hardship should not be collected and monitored as they will undoubtedly serve other purposes. For example, if the percentage of the government budget that is allocated to health remains consistently low as it does in some countries, this indicator can be a major pillar for advocacy by civil society and the international community to hold the government accountable for failing to contribute to improving people’s health. Further research on relationship between health systems financing indicators, health systems indicators and non-health systems indicators and other aspects of universal health coverage need to be undertaken in order to better develop the monitoring and evaluation framework around this key concept.

Overall, a thorough local evaluation of financial hardship and access to care based on household survey data will always be superior to any proxy indicators. As such, there is still an immense need to continually undertake household surveys, which provide invaluable information on these topics. It is only in the absence of household survey data that proxy indicators should be considered. To this end, household surveys in all countries should be strengthened and streamlined even further in order to have gold-standard information on progress towards universal health coverage [17].

Finally, direct indicators of financial hardship such as catastrophic health expenditure and impoverishment are also limited in themselves. The inclusion of financial hardship in universal health coverage is related to trying to understand and limit the health sector’s adverse economic effect on other essential components of well-being. For example if a household has to spend on health in lieu of spending on adequately on essential education, the health sector must take responsibility. Direct indicators of financial hardship may be able to capture these types of short-run events to a large extent, although as shown in this analysis, certain indicators seem to perform better than others. However, these measures do not capture long term and more drastic adverse economic impacts. These types of impacts could include splitting up of households or substitution of children’s education with employment [46]. Given the heterogeneity of these potential adverse outcomes, quantitative indicators are not best suited monitor these trends, particularly at the global level. As such, for truly understanding the adverse financial and economic impact of heath payments, the problem should be contextualized to different country settings and quantitative indicators should be supplemented with qualitative information [32].

## Additional file

Additional file 1: Table S1. Sample size (households) for each country. **Table S2.** Spearman correlation test of household variables and outcome variables. **Figure S1.** Plots of national level continuous variables against mean incidence of catastrophic health expenditure at various thresholds. **Table S3.** Sensitivity analysis of regression models – different thresholds of catastrophic health expenditure. **Table S4** – Diagnostic data for different models. **Table S5** – Correlation coefficients of national level variables.
