# Supplementary material for: Inputs for universal health coverage: a methodological contribution to finding proxy indicators for financial hardship due to health expenditure
Source: BMC Health Serv Res. 2014 Nov 25;14:577. doi: 10.1186/s12913-014-0577-2 (PMC4247877; doi:10.1186/s12913-014-0577-2)
Supplement: Additional file 1: Table S1. — Sample size (households) for each country. Table S2. Spearman correlation test of household variables and outcome variables. Figure S1. Plots of national level continuous variables against mean incidence of catastrophic health expenditure at various thresholds. Table S3. Sensitivity analysis of regression models – different thresholds of catastrophic health expenditure. Table S4 – Diagnostic data for different models. Table S5 – Correlation coefficients of national level variables. [file 12913_2014_577_MOESM1_ESM.doc]

Additional file 1

Table S1: Sample size (households) for each country

| Country | Sample Size |
| --- | --- |
| Bangladesh | 5925 |
| Bosnia and Herzegovina | 840 |
| Brazil | 4968 |
| Burkina Faso | 4928 |
| Chad | 4692 |
| China | 3990 |
| Comoros | 1824 |
| Congo | 2873 |
| Côte d'Ivoire | 3147 |
| Croatia | 988 |
| Czech Republic | 807 |
| Dominican Republic | 4930 |
| Ecuador | 4289 |
| Estonia | 992 |
| Ethiopia | 4272 |
| Georgia | 2753 |
| Ghana | 4055 |
| Guatemala | 4744 |
| Hungary | 1389 |
| India | 10478 |
| Kazakhstan | 4497 |
| Kenya | 4589 |
| Lao People's Dem. Republic | 4968 |
| Latvia | 880 |
| Malawi | 5469 |
| Malaysia | 6080 |
| Mali | 3791 |
| Mauritania | 3382 |
| Mauritius | 3960 |
| Mexico | 38482 |
| Morocco | 4996 |
| Myanmar | 6045 |
| Namibia | 4233 |
| Nepal | 8788 |
| Pakistan | 6437 |
| Paraguay | 5264 |
| Philippines | 10068 |
| Russian Federation | 3632 |
| Senegal | 3126 |
| Slovakia | 1708 |
| Slovenia | 659 |
| South Africa | 2363 |
| Spain | 5685 |
| Sri Lanka | 6775 |
| Swaziland | 2790 |
| Tunisia | 5111 |
| Ukraine | 2600 |
| United Arab Emirates | 1169 |
| Uruguay | 2970 |
| Viet Nam | 4170 |
| Zambia | 4135 |

Table S2: Spearman correlation test of household variables and outcome variables

|  | All households | | | | | | | Households with OOP >0 | | | | | | |
| --- | --- | --- | --- | --- | --- | --- | --- | --- | --- | --- | --- | --- | --- | --- |
|  | 1A - 20% | 1A - 40% | 1B - 20% | 1B - 40% | 2A | 2B | 2C | 1A - 20% | 1A - 40% | 1B - 20% | 1B - 40% | 2A | 2B | 2C |
| Household member under 5 (0,1) | 0.057 ***** | 0.052 ***** | 0.063 ***** | 0.058 ***** | 0.057 ***** | 0.037 ***** | 0.034 ***** | 0.039 ***** | 0.04 ***** | 0.032 ***** | 0.035 ***** | 0.063 ***** | 0.035 ***** | 0.029  ***** |
| Household member over 60 (0,1) | 0.084 ***** | 0.08 ***** | 0.093 ***** | 0.089 ***** | 0.02 ***** | 0.024 ***** | 0.035 ***** | 0.096 ***** | 0.089 ***** | 0.103 ***** | 0.097 ***** | 0.018 ***** | 0.021 ***** | 0.038  ***** |
| Household head with less than primary education (0,1) | 0.04 ***** | 0.062 ***** | 0.087 ***** | 0.121 ***** | 0.047 ***** | 0.02 ***** | 0.034 ***** | 0.113 ***** | 0.121 ***** | 0.111 ***** | 0.129 ***** | 0.074 ***** | 0.04 ***** | 0.061  ***** |
| Household head with primary education (0,1) | −0.028 ***** | −0.04 ***** | −0.06 ***** | −0.076 ***** | −0.001 | 0.017 ***** | 0.001 | −0.03 ***** | −0.047 ***** | −0.032 ***** | −0.052 ***** | 0.002 | 0.027 ***** | 0.003 |
| Household head with secondary or higher education (0,1) | −0.012 ***** | −0.024 ***** | −0.029 ***** | −0.05 ***** | −0.054 ***** | −0.044 ***** | −0.041 ***** | −0.089 ***** | −0.078 ***** | −0.085 ***** | −0.081 ***** | −0.084 ***** | −0.074 ***** | −0.071  ***** |
| Insurance coverage reported (0,1) | 0.005 ** | 0.004 ** | 0.006 ** | 0.003 | 0.023 ***** | 0.018 ***** | 0.003 | −0.015 ***** | −0.009 ***** | −0.007 **** | −0.003 | 0.026 ***** | 0.018 ***** | −0.003 |
| Urban residence (0,1) | −0.041 ***** | −0.057 ***** | −0.07 ***** | −0.093 ***** | −0.066 ***** | −0.03 ***** | −0.043 ***** | −0.091 ***** | −0.097 ***** | −0.077 ***** | −0.089 ***** | −0.093 ***** | −0.046 ***** | −0.064  ***** |
| Insurance coverage reported (0,1) | −0.051 ***** | −0.056 ***** | −0.082 ***** | −0.09 ***** | −0.062 ***** | −0.044 ***** | −0.035 ***** | −0.068 ***** | −0.071 ***** | −0.072 ***** | −0.076 ***** | −0.08 ***** | −0.055 ***** | −0.043  ***** |
| Disabled household member (0,1) | 0.08 ***** | 0.059 ***** | 0.076 ***** | 0.054 ***** | 0.032 ***** | 0.04 ***** | 0.037 ***** | 0.041 ***** | 0.029 ***** | 0.043 ***** | 0.032 ***** | 0.022 ***** | 0.029 ***** | 0.022  ***** |
| Quintile 1 (0,1) | −0.019 ***** | 0.008 ***** | −0.003 | 0.032 ***** | 0.002 | −0.07 ***** | −0.085 ***** | 0.128 ***** | 0.115 ***** | 0.074 ***** | 0.062 ***** | 0.043 ***** | −0.061 ***** | −0.08  ***** |
| Quintile 2 (0,1) | 0.025 ***** | 0.025 ***** | −0.007 ***** | −0.006 **** | 0.041 ***** | 0.012 ***** | 0.104 ***** | 0.094 ***** | 0.07 ***** | 0.039 ***** | 0.019 ***** | 0.07 ***** | 0.032 ***** | 0.162  ***** |
| Quintile 3 (0,1) | 0.023 ***** | 0.005 *** | 0.003 | −0.011 ***** | 0.033 ***** | 0.038 ***** | 0.096 ***** | 0.029 ***** | 0.003 | 0.013 ***** | −0.005 * | 0.042 ***** | 0.048 ***** | 0.126  ***** |
| Quintile 4 (0,1) | −0.016 ***** | −0.034 ***** | −0.002 | −0.021 ***** | −0.028 ***** | 0.03 ***** | −0.044 ***** | −0.079 ***** | −0.082 ***** | −0.031 ***** | −0.034 ***** | −0.049 ***** | 0.024 ***** | −0.074  ***** |
| Quintile 5 (0,1) | −0.013 ***** | −0.005 ** | 0.01 ***** | 0.005 ** | −0.049 ***** | −0.01 ***** | −0.071 ***** | −0.14 ***** | −0.081 ***** | −0.078 ***** | −0.031 ***** | −0.09 ***** | −0.046 ***** | −0.125  ***** |

+ rho >0.

- rho <0.

* p-value <0.1.

** p-value <0.05.

*** p-value <0.01.

***** p-value <0.005.

****** p-value <0.001.

Figure S1: Plots of national level continuous variables against mean incidence of catastrophic health expenditure at various thresholds

**
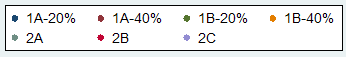
**


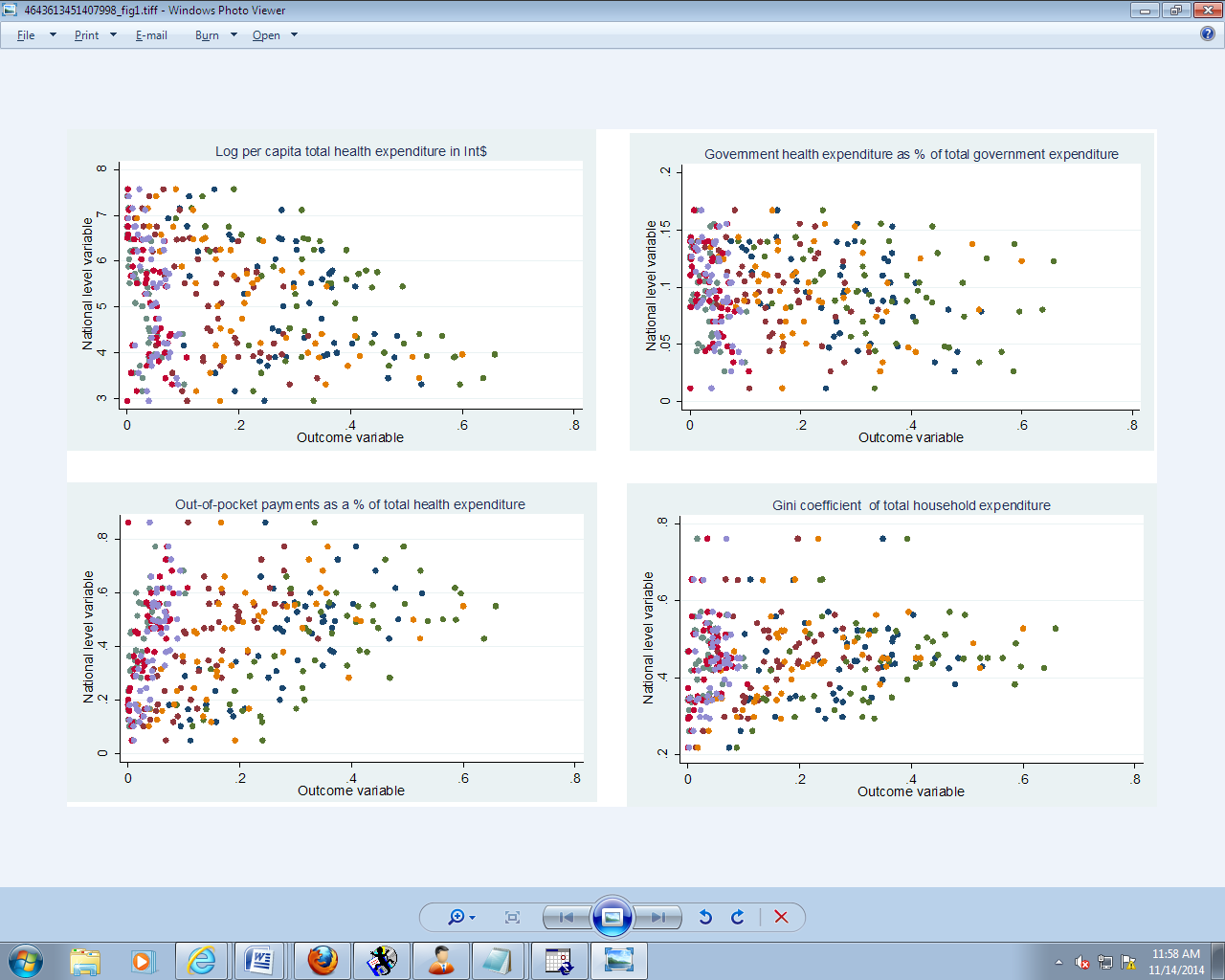


Table S3: Sensitivity analysis of regression models – different thresholds of catastrophic health expenditure

|  | All households | | | | Households with OOP >0 | | | |
| --- | --- | --- | --- | --- | --- | --- | --- | --- |
|  | 1A - 10% | 1A - 30% | 1B - 10% | 1B - 30% | 1A - 10% | 1A - 30% | 1B - 10% | 1B - 30% |
| National level variables |  | | | | | | | |
| Per capita total health expenditure (PPP $) | 0.095 | −0.046 | 0.046 | 0.025 | 0.031 | 0.001 | −0.047 | −0.029 |
| Government health expenditure over total government expenditure (%) | −1.400 ***** | −0.235 | −1.307 | −0.136 | 0.630 | 2.625 | 3.566 | 2.508 |
| Gini coefficient | 0.331 | 1.224 ** | 1.522 ***** | 2.01 **** | 1.659 ** | 1.411 ** | 2.411 **** | 1.677 ** |
| Predominately tax-funded financing (0,1) | 0.204 | −0.050 | 0.086 | −0.169 | −0.050 | −0.072 | −0.005 | −0.119 |
| Predominately mixed financing (0,1) | 0.158 | −0.011 | −0.045 | −0.179 | 0.031 | 0.101 | −0.029 | −0.091 |
| Out-of-pocket payments as a share of total health expenditure (%) | 1.642 ***** | 2.307 ***** | 1.539 ***** | 2.666 ***** | 2.265 ***** | 2.105 ***** | 2.399 ***** | 2.658 ***** |
| Household level variables |  |  |  |  |  |  |  |  |
| Household member under 5 (0,1) | 0.291 ***** | 0.224 ***** | 0.262 ***** | 0.16 ***** | 0.002 | 0.006 | 0.015 | −0.003 |
| Household member over 60 (0,1) | 0.343 ***** | 0.363 ***** | 0.359 ***** | 0.384 ***** | 0.353 ***** | 0.328 ***** | 0.382 ***** | 0.357 ***** |
| Household head with primary education (0,1) | 0.036 | −0.062 ** | −0.015 | −0.132 ***** | −0.072 *** | −0.177 ***** | −0.109 ***** | −0.205 ***** |
| Household head with secondary or higher education (0,1) | −0.072* | −0.269 ***** | −0.110 **** | −0.308 ***** | −0.202 ***** | −0.388 ***** | −0.255 ***** | −0.407 ***** |
| Male household head (0,1) | −0.032 | −0.083 ***** | −0.030 | −0.07 **** | −0.123 ***** | −0.129 ***** | −0.098 ***** | −0.104 ***** |
| Urban residence (0,1) | −0.121 **** | −0.249 ***** | −0.182 ***** | −0.291 ***** | −0.118 ***** | −0.272 ***** | −0.132 **** | −0.267 ***** |
| Insurance coverage reported (0,1) | −0.080 * | −0.156 ***** | −0.110 ** | −0.195 ***** | −0.104 ***** | −0.166 ***** | −0.115 ***** | −0.18 ***** |
| Disabled household member (0,1) | 0.576 ***** | 0.548 ***** | 0.528 ***** | 0.462 ***** | 0.295 ***** | 0.313 ***** | 0.284 ***** | 0.289 ***** |
| Quintile 2 (0,1) | 0.315 ***** | 0.146 ***** | 0.111 ** | −0.08 * | −0.313 ***** | −0.284 ***** | −0.275 ***** | −0.251 ***** |
| Quintile 3 (0,1) | 0.348 ***** | 0.063 | 0.158 *** | −0.091 | −0.681 ***** | −0.593 ***** | −0.469 ***** | −0.375 ***** |
| Quintile 4 (0,1) | 0.315 ***** | −0.059 | 0.224 **** | −0.037 | −1.130 ***** | −0.926 ***** | −0.688 ***** | −0.447 ***** |
| Quintile 5 (0,1) | 0.288 ***** | 0.054 | 0.247 **** | 0.061 | −1.473 ***** | −0.954 ***** | −0.981 ***** | −0.468 ***** |

Table S4 – Diagnostic data for different models

|  | All households | | | Households with OOP>0 | | |
| --- | --- | --- | --- | --- | --- | --- |
|  | AIC | BIC | LogLik | AIC | BIC | LogLik |
|  | 1A-20 | | | 1A-20 | | |
| RI.MACRO | 284153.0 | 284236.1 | −142068.5 | 181014.0 | 181092.7 | −90499.0 |
| RI.MICRO | 279215.2 | 279360.7 | −139593.6 | 171688.2 | 171825.9 | −85830.1 |
| RI.MACRO+MICRO | 279197.9 | 279405.8 | −139578.9 | 171665.8 | 171862.5 | −85812.9 |
| RC.MACRO+MICRO | 275411.0 | 276554.5 | −137595.5 | 170085.9 | 171167.9 | −84933.0 |
|  | 1A-40 | | | 1A-40 | | |
| RI.MACRO | 211940.2 | 212023.3 | −105962.1 | 158842.1 | 158920.8 | −79413.0 |
| RI.MICRO | 207225.5 | 207371.1 | −103598.8 | 151612.2 | 151749.9 | −75792.1 |
| RI.MACRO+MICRO | 207202.6 | 207410.5 | −103581.3 | 151584.5 | 151781.2 | −75772.3 |
| RC.MACRO+MICRO | 204719.0 | 205862.5 | −102249.5 | 150009.5 | 151091.5 | −74894.8 |
|  | 1B-20 | | | 1B-20 | | |
| RI.MACRO | 303672.8 | 303756.0 | −151828.4 | 174960.0 | 175038.7 | −87472.0 |
| RI.MICRO | 298916.8 | 299062.4 | −149444.4 | 170300.6 | 170438.3 | −85136.3 |
| RI.MACRO+MICRO | 298900.6 | 299108.5 | −149430.3 | 170278.1 | 170474.8 | −85119.0 |
| RC.MACRO+MICRO | 294602.5 | 295746.0 | −147191.2 | 169205.0 | 170286.9 | −84492.5 |
|  | 1B-40 | | | 1B-40 | | |
| RI.MACRO | 248298.2 | 248381.3 | −124141.1 | 167329.3 | 167408.0 | −83656.7 |
| RI.MICRO | 242950.8 | 243096.3 | −121461.4 | 162835.6 | 162973.3 | −81403.8 |
| RI.MACRO+MICRO | 242926.3 | 243134.2 | −121443.1 | 162808.9 | 163005.6 | −81384.4 |
| RC.MACRO+MICRO | 239068.8 | 240212.3 | −119424.4 | 161626.5 | 162708.4 | −80703.2 |
|  | 2A | | | 2A | | |
| RI.MACRO | 68326.2 | 68409.4 | −34155.1 | 59044.1 | 59122.8 | −29514.1 |
| RI.MICRO | 65557.9 | 65703.4 | −32765.0 | 55657.4 | 55795.1 | −27814.7 |
| RI.MACRO+MICRO | 65537.3 | 65745.3 | −32748.7 | 55630.9 | 55827.6 | −27795.4 |
| RC.MACRO+MICRO | 60571.8 | 61715.3 | −30175.9 | 50545.5 | 51627.5 | −25162.8 |
|  | 2B | | | 2B | | |
| RI.MACRO | 84696.8 | 84780.0 | −42340.4 | 72170.6 | 72249.3 | −36077.3 |
| RI.MICRO | 81216.9 | 81362.4 | −40594.4 | 69901.2 | 70038.9 | −34936.6 |
| RI.MACRO+MICRO | 81205.9 | 81413.8 | −40582.9 | 69886.6 | 70083.3 | −34923.3 |
| RC.MACRO+MICRO | 73632.7 | 74776.2 | −36706.4 | 62123.3 | 63205.3 | −30951.7 |
|  | 2C | | | 2C | | |
| RI.MACRO | 97627.8 | 97710.9 | −48805.9 | 83142.8 | 83221.5 | −41563.4 |
| RI.MICRO | 89186.5 | 89332.1 | −44579.3 | 74358.5 | 74496.2 | −37165.3 |
| RI.MACRO+MICRO | 89173.9 | 89381.8 | −44567.0 | 74345.3 | 74542.1 | −37152.7 |
| RC.MACRO+MICRO | 79858.6 | 81002.1 | −39819.3 | 65004.8 | 66086.8 | −32392.4 |

RI.MACRO: random intercept model for country effects.

RI.MICRO: random intercept model for country effects with just household level variables.

RI.MACRO+MICRO: intercept model for country effects with both national and household variables.

RC.MACRO+MICRO: random coefficients model with varying coefficients for household variables at the country level alongside national level variables and random country intercepts.

Table S5 – Correlation coefficients of national level variables

|  | Log of per capita total health expenditure (PPP $) | Government health expenditure as % of total government expenditure (%) | Out-of-pocket payments as a % of total health expenditure (%) | Gini coefficient of total household expenditure | Predominately tax-funded financing (0,1) | Predominately mixed financing (0,1) |
| --- | --- | --- | --- | --- | --- | --- |
| Log of per capita total health expenditure (PPP $) | 1 |  |  |  |  |  |
| Government health expenditure as % of total government expenditure (%) | 0.4466 | 1 |  |  |  |  |
| Out-of-pocket payments as a % of total health expenditure (%) | −0.5025 | −0.5106 | 1 |  |  |  |
| Gini coefficient of total household expenditure | −0.2959 | −0.1062 | 0.0722 | 1 |  |  |
| Predominately tax-funded financing (0,1) | −0.4923 | −0.5016 | 0.1621 | 0.354 | 1 |  |
| Predominately mixed financing (0,1) | 0.0332 | 0.2121 | 0.3029 | 0.0856 | −0.5259 | 1 |
